# Exploring how the design and provision of digital self-management technology can improve the uptake by older adults with chronic kidney disease, diabetes and dementia: A modified e-Delphi study

**DOI:** 10.1177/20552076241247196

**Published:** 2024-06-07

**Authors:** Louise Moody, Esme Wood, Abigail Needham, Andrew Booth, Wendy Tindale

**Affiliations:** 1 Centre for Arts, Memory and Communities, 2706 Coventry University, UK; 2 NIHR Devices for Dignity HealthTech Research Centre, 7318 Sheffield Teaching Hospitals NHS Foundation Trust, UK; 3 School of Health and Related Research (ScHARR), 7315 University of Sheffield, Sheffield, UK

**Keywords:** Digital technology, self-management, older adults, diabetes, chronic kidney disease, dementia

## Abstract

**Objectives:** As development and introduction of digital self-management technologies continues to increase, the gap between those who can benefit, and those who cannot correspondingly widens. This research aimed to explore the use of digital self-management technology by older adults with three highly-prevalent long-term conditions (chronic kidney disease, diabetes and dementia), and build expert consensus across the conditions on changes needed to improve effective usage. **Method:** This qualitative research involved a modified e-Delphi Study. The Delphi panel was comprised of experts with personal, academic or clinical expertise related to one of the long-term conditions and/or the development and use of digital self-management technology. The e-Delphi involved a round of online semi-structured interviews followed by two rounds of a structured online survey. **Results:** Fourteen experts participated in the study, with eleven of the fourteen completing all three rounds. Analysis of the interviews (round 1 of the Delphi) led to 7 main themes and 29 sub-themes. These were translated into 26 statements that formed the basis of the online survey questions. In the first administration of the survey (round 2) 19 statements reached consensus. After the second administration a further 6 statements reach consensus. **Conclusion:** The findings reflect expert consensus on barriers to the use of digital self-management by older adults with 3 different, but inter-related conditions, and identify ways in which the design and provision of such technologies could be improved to facilitate more effective use. It is concluded that both the design and the provision of technologies should consider a combination of individual, condition-specific and age-related requirements. By building a consensus on issues and potential strategies common across the three conditions, we aim to inform future research and practice and facilitate effective self-management by older adults.

## Introduction

Digital self-management technology has the potential to increase and improve access to support and care for older adults living at home with long-term conditions^1,2^ with technology increasingly deployed for health assessment and support, information and advice, social and intergenerational connection and engaging in healthy living behaviours (e.g. diet and exercise).^3,4,5^ The acceptance and adoption of digital technology by older adults arguably increased during the COVID-19 pandemic, as did a need for self-management and the use of digital health tools.^6–8^ While there has been a proliferation of health technologies and advances in acceptance have been made, they have not been fully adopted at scale by older adults, with continued barriers to use and a remaining digital divide.^9–12^

Understanding and improving the adoption of effective self-management technologies arguably has the potential to reduce strain on health and social care and help address inequity in health outcomes.^13,14^ Recent scoping reviews reflect a growing interest in understanding barriers and facilitators to the use of technologies by older adults for a range of conditions,^10^ for health promotion and disease prevention^15,16^ and self-regulation and autonomy in daily living.^2^ In building upon the current evidence base, our focus is specifically on the use of digital technology for the self-management of three conditions that are common among older adults (e.g. 65 years old plus)^17^: chronic kidney disease (CKD), dementia and diabetes. The self-management practices employed across these three conditions share some similarities such as the need to manage medication, follow guidelines, make decisions. Conversely, each condition imposes specific requirements on the individual, their social support network and on any supportive technology. The conditions can be inter-related with a strong potential for co-morbidity.^18,19^ Arguably, by building an understanding across these conditions, we will be better placed to design, identify and provide suitable technology to older adults living with more than one condition.

### Digital self-management technologies

Self-management involves an individual taking responsibility for the management of their long-term condition (including managing symptoms; treatments; adopting healthy habits; making lifestyle and psychosocial adjustments and reducing risk) to improve their health in collaboration with others including their family, community and healthcare professionals.^20–22^ Increasingly, digital technology plays a role, with smartphones and tablets offering functionality to support the management of chronic disease, for example through electronic records, self-monitoring and record-keeping, contact with healthcare professionals, access to information resources and support for activities of daily living.^23,24^ In the context of the three conditions of focus here, self-management technologies include but are not limited to home dialysis machines, reminder and alerts systems, GPS locating technologies, smart insulin pens and intelligent medication dispensers.

Self-management, and the use of enabling digital self-management technologies, is increasingly advocated to play a role in increasing the efficiency of and access to care; reducing the use of in-patient health and social care use and the associated costs; and improving health, well-being and independence.^22,25–27^ As well as the potential benefits to healthcare and healthcare professionals, patients may be able to maintain greater control over their care, as well as autonomy and independence (e.g. remaining in their own homes) for longer.^28^

Despite the purported benefits, peoples interest in and capacity to self-manage and take responsibility for their own health varies, as well as their willingness and capacity to use the associated technology.^22,29^ Multi-morbidity and inter-related conditions^13^ pose added challenges, including the uncertainty and knowledge of what to do in response to symptoms and the management of multiple medications and healthy behaviours.^30^ The impact on families and carers of self-management is variable and multi-dimensional, but often, caregivers play an integral role in supporting patient self-management.^31^

Various clinically effective, self-management technologies are available but are often underused, not adopted at scale, or abandoned by users.^32,33^ Unused, or incorrectly used technology can lead to poor condition management, a lower quality of life and risk of medical complications.^34^ This suboptimal condition management, wasted equipment and increased need for treatments can incur a heavy financial burden for health and care systems.^35,36^

The barriers to technology use by older adults have been well explored and include poor design; failure to meet needs and aspirations; limited involvement of end-users in the development; lack of interest and understanding of the benefits; strong anti-ageing trends and stigma; poor access and broadband services; confidence and skills gap; and the effort or behaviour change required.^1,5,37–41^ In response, organisational and academic approaches have sought to improve both the design, provision and adoption of technology offering guidance for the healthcare workforce,^42^ frameworks and toolkits to guide the design and implementation of technology,^38,40^ and support matching the users’ characteristics, environment and preferences, to the functions and features of the technology.^43^

While understanding and awareness of barriers and enablers have increased, issues remain in terms of the provision and tailoring of technology to ensure acceptance and long-term use in specific circumstances. Here we explore the use of digital self-management technology, in the context of three inter-related long-term conditions by older adults. Despite high rates of multi-morbidity, technology solutions often focus on the management of one condition.^44^ Here, we begin to consider digital technology use across three conditions,^45,46^ with the long-term goal of informing the design of technology that facilities self-management by those living with more than one long-term condition.

## Systematic reviews of qualitative research

In recognition of the existing body of literature, three separate condition-specific systematic reviews of qualitative research (qualitative evidence syntheses) were undertaken to inform our approach and consider: *What are the experiences and attitudes of older adults with chronic kidney disease, dementia or diabetes in relation to the use of technologies to support self-management?* The methods are fully detailed in a registered protocol.^47^ The syntheses were undertaken sequentially to accumulate learning across the conditions and focus on findings from the successive reviews rather than repeat common elements. The detailed findings are reported elsewhere^48^ with a summary of salient points below.

The synthesis confirmed that increasingly technology plays a role in self-management by older adults. Smartphones and tablet applications provide access to and ownership of, electronic records, self-monitoring and record-keeping, contact with the health and social care professional team, patient education and information, activity planning for daily living and devices to stimulate activity, or cognition.^23,24^ The use of technology across the three conditions is determined by multiple factors including prior knowledge, age, the nature of their health condition, their treatment, motivation, digital literacy, the support they receive communication and language skills, any age-related physical or cognitive restrictions and behavioural choices.^49^

The literature demonstrated a general assumption of empowerment through knowledge.^34,50,51^ However, it is important that the information is tailored^52^ or personalised to the specific needs of the patient,^53^ with the challenge of how to provide personalised and individually tailored information at the right time and in the right quantity to meet the needs of the individual and their carer.^53^

Older adults typically want to understand how a technology will help them. They are more likely to consider the use of a technology that supports them in a previously enjoyed activity rather than to support a new interest or activity.^54^ Trust in the technology, and in the information it provides is also critical.^53,55,56^ Patients are more likely to accept their own need and to be willing to use technology where they have the support of a wider social network who may help instil trust, encourage the use of technology and may provide instruction or technical support.^57^ The research suggests motivation is greater if a family member has purchased the technology, or if enables communication or activity with them.^58^ Across the conditions the three reviews suggest there are significant limitations when targeting technology only at the older adult as the primary user, given the influence of wider support and the clinical and family environment in which it is often used.^57^

Limited consideration of the gerontological aspects affecting usage^24^ was found, with for example, little consideration of the *functional* difficulties of use related to ageing such as manual dexterity and visual impairments.,^23,59^ There tends to be a stronger focus on *generational* aspects such as the extent of intuitive knowledge about how technologies operate and motivation to use new technology and learn new skills.^52^ Relatively little exploration of the inter-related nature of the conditions was also recognised. It is not uncommon for example for older adults to experience *both* CKD *and* diabetes for example,^60^ with both conditions requiring dietary management and self-monitoring.^23,61^

Having identified these common findings in the literature we aimed to explore and build upon them with a panel of experts with knowledge of the three conditions, self-management approaches and enablers and barriers to the use of digital technology by older adults. By building a consensus on issues and potential strategies common across these inter-related conditions, we aimed to inform future research and practice and facilitate effective self-management.

## Methods

Ethical approval was provided by the Coventry University Research Ethics Committee (P124976). The Delphi technique^62^ was selected as a method of collating expert views and building consensus. The three conditions are of interest to the wider NIHR Devices for Dignity HealthTech Research Centre (D4D) through which the authors collaborate, and the method was chosen as way to draw together expert views on areas of future development and research.

### Planning and design

Building on the knowledge gained through the reviews, the Delphi study was designed to explore the common themes from the combined synthesis. The method seeks to achieve consensus among an expert panel through a multi-stage, systematic collection and aggregation of views.^62,63^ Here, consensus was employed to determine if agreement existed among the panel of experts representing different conditions and as a stopping guideline on the number of rounds.

Undertaken during 2021, when measures in response to the COVID-19 pandemic affected in-person data collection and collaboration (particularly for those living with and working with those living with long-term conditions), a modified e-Delphi method was used. This involved online interviews and the use of an online survey platform to collect data and provide a structure by which to reach consensus. This approach allowed participants to respond remotely, at times convenient to them, which was particularly necessary for the healthcare professionals. The data collection was undertaken by an independent researcher (EW) who is not supported directly by D4D. The researcher's clinical training and experience gave her a sound knowledge of all three conditions. The data collection materials were piloted locally among colleagues ahead of use within the study and adjusted to improve clarity as required.

### Participants

A heterogeneous panel was sought to provide opinions from different perspectives and was achieved through purposive sampling. They were selected based on meeting one of the following criteria:

- an older person (55+) with direct lived experience of one of the three conditions.
- a current HCPC or NMC registered health professional with experience of working as a NHS grade 7 specialist in the field of one or more of the conditions.
- a developer/designer who had a track record of developing multiple successful platforms or products specifically for the needs of either older people or people with one of the three conditions.
- a recognised academic with specialist knowledge of this area at associate professor level or above and publication record to support this.

Potential panel members were identified through academic and clinical publications and events and through the D4D academic and clinical network. Participants were approached by email and invited to join the panel (by EW). The study was explained and participant information provided. For those that responded to the email, additional study information was provided through a telephone call with the opportunity to ask questions and seek clarification. Fully informed, written consent was obtained. The participants remained anonymous to each other throughout the data collection rounds to encourage the expression of an unbiased opinion.

### Procedure and data collection

**Figure 1.**
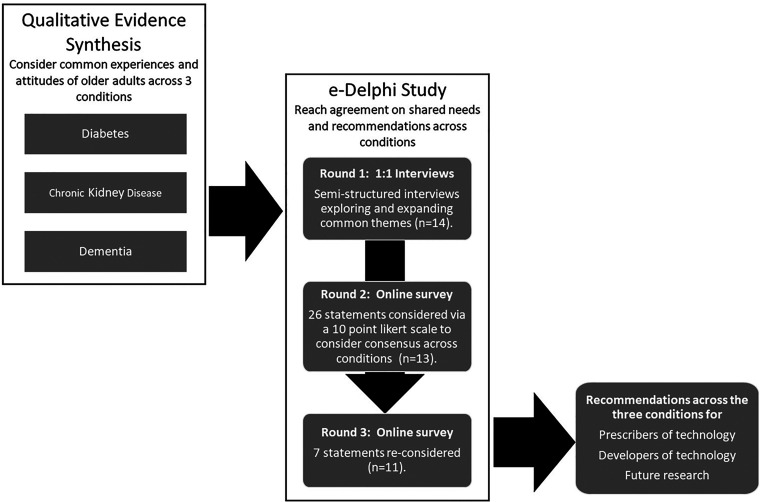
An overview of the method and stages of the modified e-Delphi process.

The stages of the modified e-Delphi process are outlined below in Figure 1.

#### Round 1: exploration and expansion of common themes

The first round of qualitative data collection involved online interviews (using Zoom). The interviews were guided by a set of semi-structured questions informed by the findings of the qualitative evidence synthesis (see Moody et al. 2022). The questions prompted the panellists to respond to and expand upon the themes emerging from the literature based on their own expertise and consider additional challenges and barriers in the use of self-management technology and requirements for improved technology design and provision.

Automatically generated transcripts were downloaded from Zoom, checked against the audio recording and edited for accuracy. Inductive thematic analysis was undertaken.^64^ Themes were highlighted within each transcript then grouped across transcripts to identify commonalities, differences and patterns. The interviews were analysed first by long-term condition, to ensure concepts and condition-specific data were not missed for each condition. Then to gain a ‘whole picture’ view, themes and sub-themes across the whole data set were generated.

#### Round 2: consensus on needs and barriers across conditions

Round 2 of the Delphi method involved a structured online survey. The survey was hosted on an online survey platform (www.onlinesurveys.ac.uk/). A link to the survey was distributed to all of the original panellists from round 1 via email. The survey consisted of 26 statements derived from the key messages identified through the thematic analysis of the interview data in round 1. The survey employed a Likert scale and respondents were asked to individually rate each statement with a score of 1–10 (1 = strongly disagree, 10 = strongly agree) to indicate the extent to which they agreed with it. A 10 point survey was chosen for ease of use and to enable a wider expression of views, while avoiding a midpoint that enables a neutral response rather than a clear decision.^65,66^

The results were collated and a mean and standard deviation were calculated for each statement as well as the variance, as a measure of variability or spread of the responses (the average of squared deviations from the mean). Agreement with the statement was considered to be reflected by rating or mean of 6 or more, strong agreement was considered to be represented by a rating of 8 or more. Consensus was defined as a variance of less than 4 and therefore no consensus by a variance of greater than 4.

#### Round 3: final consensus on needs and barriers

In the third round of data collection, the seven statements identified from round 2, which had not reached consensus were presented back to the panel via a second online survey. This approach was chosen to shorten the survey, aiming to reduce attrition, but not to influence the overall importance of the statements.^66^ Panellists again rated the statements using the same 1–10 Likert scale. They were also provided with the mean rating for each statement from the previous round, to allow them to re-consider their view in the context of the wider panel views. The results were collated and a mean, standard deviation and the variance for each statement calculated.

## Results

During June to December 2021, a panel of experts participated in the e-Delphi study, to reach consensus on common issues and approaches to improving or encouraging further use and uptake of digital self-management technology by older adults with chronic kidney disease, diabetes and dementia.

### Panel characteristics

Fourteen experts participated, with 11 of the 14 completing all three rounds. The expertise of the panel is summarised in Table 1. There was a reasonably equal balance of expertise across the three clinical conditions. The panel included three participants with lived experience of the conditions (two living with diabetes, one with CKD); the panel did not include a participant living with dementia. As can be seen in Table 1, participants had a combination of expertise (e.g. lived experience and academic, clinical and academic), leading to 6 participants with experience as healthcare professionals, 9 academics and 2 with expertise in the development of self-management technology.

**Table 1.** Matrix of panel expertise (*n* = 14).

Expertise/specialism	Number of participants	Participant number (shaded grey indicates the expertise)													
		1	2	3	4	5	6	7	8	9	10	11	12	13	14
Dementia	6	✓	✓	✓	✓	✓								✓	
Diabetes	7					✓	✓	✓	✓	✓	✓			✓	
CKD	5					✓						✓	✓	✓	✓
Technology developer	2					✓								✓	
Academic	9	✓	✓	✓	✓	✓	✓	✓		✓	✓		✓		
Health Professional	6			✓	✓		✓	✓					✓		✓
Expert through lived experience	3								✓		✓	✓			
**Total**	**14**														

#### Round 1: exploration and expansion of common themes across conditions

The interview data generated in round 1 was initially analysed by condition, with themes and sub-themes generated for each condition. Then to gain a view across the three long-term conditions, the themes and sub-themes across the whole data set were combined and grouped in overall themes. These sub-themes were re-read in the context of their original interviews and regrouped, with similar sub-themes being merged and new overall themes being formed. These themes are summarised in Table 2 and were used to inform the design of the online survey deployed in round

**Table 2.** Interview themes across panellists and conditions.

	Interview theme	Sub-themes
**1**	How is self-management technology used by older adults with long-term health conditions?	• Coaching, goal setting and social support • Patient education • Treatment planning and data sharing with the clinical team • Self-monitoring • Prompts and reminders • Passive technologies • Used by family carers
**2**	The key barriers to the use of self-management technologies by older adults with long-term health conditions.	• Digital skills and literacy • Lack of access to digital technology infrastructure • Impact of poverty • Lack of diversity
**3**	Assessing individual need to select self-management technologies for use by older adults with long-term health conditions.	• Individualised approach to technology provision • Ongoing assessment • Technology abandonment • How do you measure success?
**4**	Understanding the right support needed for older adults with long-term health conditions to use self-management technologies effectively.	• Support to start using new technologies • The needs for ongoing support • Supporting family carers
**5**	A role for multi-functional self-management technologies for older adults with long-term health conditions.	• One system with multiple functions • The burden of data entry to users • Future developments • Not all data is helpful
**6**	Understanding trust, privacy and data sharing in self-management technologies for older adults with long term-health conditions.	• Data Sharing with health professionals • Protecting privacy and control of data sharing • Acceptance and trust
**7**	Self-management technologies for older people with long-term health conditions should be both accessible and aspirational in design.	• Technologies not designed for older adults • Stigma • Designing for additional impairments • Aspirational design

#### Round 2: consensus across conditions

The survey administered in round 2 asked respondents to indicate their agreement to 26 statements derived from the key messages and themes from the round 1 interviews. The survey had a 92% response rate (completed by 13 of the original 14 expert panel members). The results, in term of the mean rank, variance and standard deviation calculated for each statement are detailed in Table 3.

**Table 3.** Delphi survey results (rounds 2 and 3) (those labelled in bold indicate no consensus reached (variance = >4)*.*

		Round 2			Round 3		
	Statements	Mean	SD	Variance	Mean	SD	Variance
2.3	Technology development, research and provision in the UK do not actively seek to include a diverse and representative group of users. E.g. the use of English language only software, or exclusion of those whose condition has progressed or become more complex with age.	7.91	0.67	0.45			
4.2	The initial setting up or on boarding of users is crucial to the person's sense of trust in the technology.	9.31	0.82	0.67			
6.5	Control over who accesses data from self-management technologies should rest with the individual user.	8.62	1.15	1.31			
3.1	Rather than a ‘one size fits all’ solution, successful self-management technologies need to be designed to be adaptable, so that they can easily be incorporated into the lives and homes of older adults with long-term health conditions.	9.15	1.17	1.36			
6.2	There are many benefits from using technology to share personal health data with the clinical team, such as promoting greater collaborative management, improvement in communication and informing day-to-day decision-making for both the user and clinical team.	8.15	1.17	1.36			
3.3	Assessment should also be ongoing, with recognition of a person's changing needs or wishes, supporting the use of self-management technologies to enable the user.	9.23	1.25	1.56			
5.3	The ability to personalise technology set up may limit the potential data entry burden on users by only allowing appropriate data to be recorded that supports and enables the user in managing their condition or day-to-day lives.	7.67	1.25	1.56			
6.1	Older adults seek reassurance from organisations such as the NHS or local authority, that the apps, technologies and wearables they have access to are both appropriate for the intended use and trustworthy.	7.92	1.27	1.61			
3.4	Success of self-management technologies is often measured through its impact on clinical services, sales figures or reduced carer burden.	7.83	1.28	1.64			
4.3	For older adults, self-management technologies are sometimes used within the supportive environment of their family carers, where their ability to input data or use technologies is dependent upon the active involvement of others.	7.92	1.44	2.08			
5.2	Not all users want to view and record all aspects of their health and well-being	8.23	1.53	2.33			
7.1	Self-management technologies designed to support a particular long-term condition often fail to take into consideration common disabilities associated with older age.	7.77	1.58	2.49			
6.3	Sharing of personal health data can lead to a decrease in personal responsibility for managing one's own health condition.	**4.42**	1.61	2.58	4.40	1.50	2.24
7.5	The development of aspirational self-management technologies for older adults would likely increase the uptake and use.	7.77	1.62	2.64			
2.1	The most prominent barrier to the effective use of self-management technologies by older adults with long-term health conditions is limited digital skills and confidence in using new technologies.	6.38	1.78	3.16			
4.1	Introducing self-management technologies to older adults requires a greater level of support to ensure they have the appropriate skills and knowledge to use it effectively.	8.08	1.8	3.24			
2.2	The most significant demographic factor in access to self-management technologies was the impact of poverty and the financial implications of relying on technologies that may be expensive to purchase, maintain or keep connected.	7.15	1.83	3.36			
7.4	The wearing of obviously ‘disability friendly’ trackers and pendants can increase stigma in the community and increase vulnerability.	7.54	1.87	3.48			
5.1	There is a recognised need for self-management technologies that undertake several different functional tasks to support older adults with long-term conditions.	6.42	1.89	3.58			
6.6	If user privacy is both valued and respected they are more likely to opt to share data more widely in instances where it may be beneficial. For example, with online support groups who can offer peer feedback, motivation and encouragement in response.	8.00	1.91	3.67			
7.2	Technologies that are designed specifically for older adults are often unappealing, clinical or stigmatising.	7.77	2.04	**4.18**	7.60	1.43	2.04
6.4	Sharing of personal health data can raise expectations about the degree to which the clinical team will monitor and respond to data being shared	6.67	2.05	**4.22**	6.60	1.74	3.04
7.3	Few older adults are keen to have equipment on display in their homes that looks clinical.	7.23	2.08	**4.33**	7.50	1.36	1.85
3.2	Self-management technologies need to be provided in a customised and individualised way, following detailed holistic assessment of the individual's needs and wishes.	8.54	2.1	**4.40**	8.10	2.21	**4.89**
1.2	Technologies designed for self-management are often used in partnership with family carers or on behalf of people with long-term conditions by their carers	6.75	2.2	**4.85**	6.30	1.79	3.21
1.1	Older adults with long-term conditions use self-management technology in a variety of different ways, seeking to undertake a range of functional tasks based on their individual needs.	7.83	2.23	**4.97**	7.60	1.28	1.64

The mean results indicate that the panellists agreed with most of the statements presented, with only one statement having a mean rating below 6 (statement 6.3 *Sharing of personal health data can lead to a decrease in personal responsibility for managing one's own health condition*). Of the 26 statements, 19 reached consensus after round 2, suggesting a strong agreement across the panel at an early stage. These statements did not need to be considered further in the third round of data collection. There were 7 (statements 1.1, 1.2, 3.2, 6.4, 7.2, 7.3), where the variance of panellists ratings exceeded 4, indicating a greater variation in opinion. These statements were continued into the third round to determine if agreement could be reached among the panel.

#### Round 3: final consensus

A 78% response rate was achieved in round 3 (11 of the original 14 panel members). The mean rank, variance and standard deviation for each statement are reported in Table 3. Of the 7 statements that were re-considered in the third round, consensus was reached on 6.

### Non-consensus and no agreement

The panellists did not agree (mean 4.4) with statement 6.3 (*Sharing of personal health data can lead to a decrease in personal responsibility for managing one's own health condition*. There was no consensus (variance 4.89) reached for statement 3.2 (*Self-management technologies need to be provided in a customised and individualised way, following detailed holistic assessment of the individual's needs and wishes)*. There was a clear difference of opinion and so this is not considered as a generalisable finding across conditions. The interview data suggested that panellists with expert knowledge in the use of self-management technologies for people with dementia advocate for the importance of this issue, more so for those considering the use of self-management technologies by older adults with CKD and/or diabetes.

### Agreement across conditions

The statements provoking the highest ratings (a mean of 8+), or agreement with the statement from the panel and reaching consensus in round 2 were:

- 4.2. The initial setting up or on boarding of users is crucial to the person's sense of trust in the technology (mean 9.31, SD 0.82).
- 3.3. Assessment should also be ongoing, with recognition of a person's changing needs or wishes, supporting the use of self-management technologies to enable the user (mean 9.23, SD 1.25).
- 3.1. Rather than a ‘one size fits all’ solution, successful self-management technologies need to be designed to be adaptable, so that they can easily be incorporated into the lives and homes of older adults with long-term health conditions (mean 9.15, SD 1.17).
- 6.5 Control over who accesses data from self-management technologies should rest with the individual user (mean 8.62, SD 1.15).
- 5.2. Not all users want to view and record all aspects of their health and well-being (mean 8.23, SD 1.53)
- 6.2. There are many benefits from using technology to share personal health data with the clinical team, such as promoting greater collaborative management, improvement in communication and informing day-to-day decision-making for both the user and clinical team (mean 8.15, SD 1.17).
- 4.1. Introducing self-management technologies to older adults requires a greater level of support to ensure they have the appropriate skills and knowledge to use it effectively (mean 8.08, SD 1.8).
- 6.6. If user privacy is both valued and respected they are more likely to opt to share data more widely in instances where it may be beneficial. For example, with online support groups who can offer peer feedback, motivation and encouragement in response (8.0, SD 1.91).

There was highest consensus for the following statement:

- 2.3. Technology development, research and provision in the UK do not actively seek to include a diverse and representative group of users. (e.g. the use of English language only software, or exclusion of those whose condition has progressed or become more complex with age) (mean 7.91, SD 0.67).

**Figure 2.**
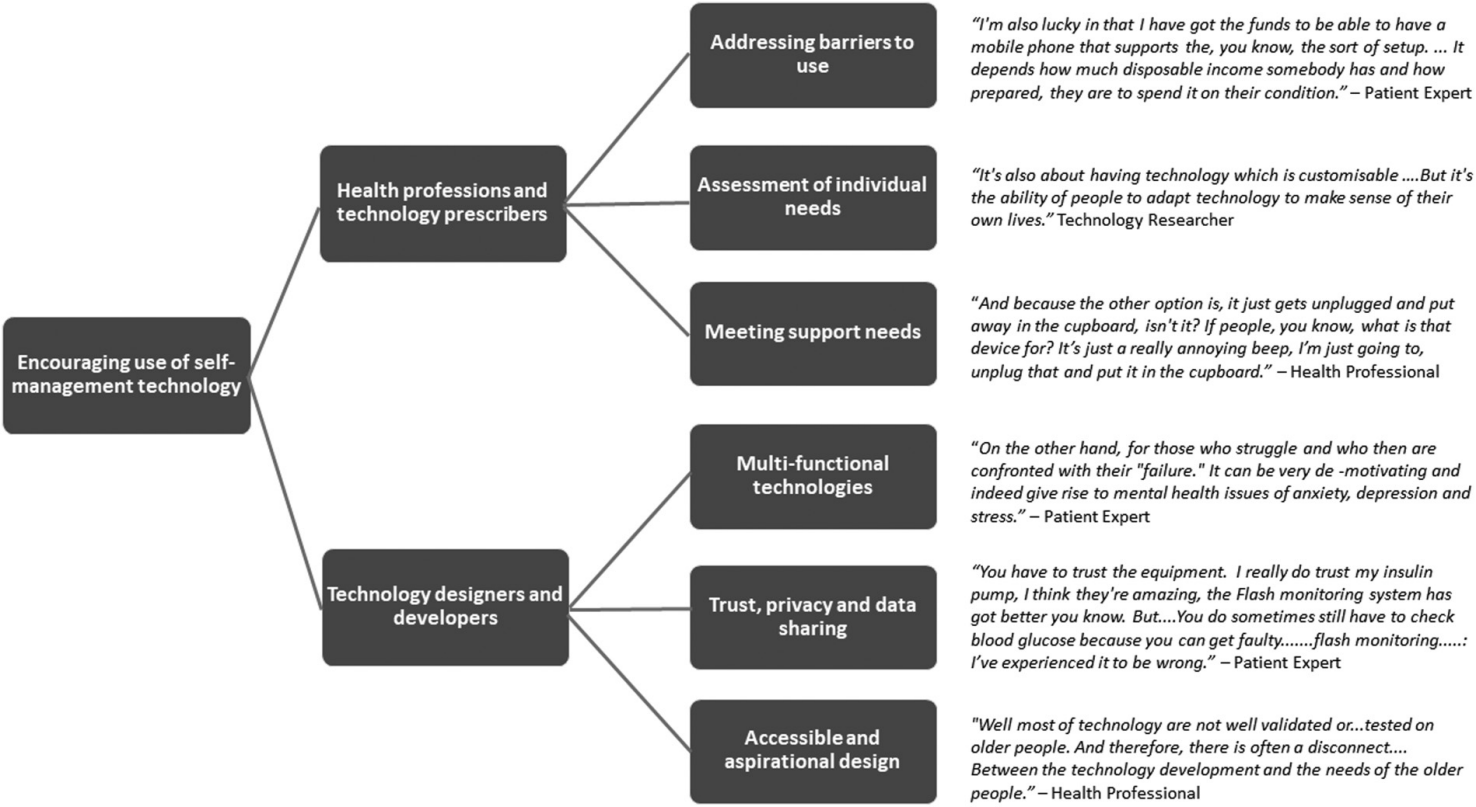
A summary of areas to be addressed through the technology design and provision.

An overview of key findings from the interviews and subsequent rounds of the Delphi are provided in Figure 2, where we summarise at a high level the emerging areas to be addressed through the design and provision of technology to older adults, with illustrative quotes from the interviews. These are expanded upon through the suggested recommendations listed in Table 4.

**Table 4.** Summary of recommendations for practitioners.

Design for…	Provision and support by healthcare professionals
*Integrated management of multiple conditions:* Older adults often have to manage more than one long-term condition. Integrated solutions, which reduce the number of applications used and manage the inter-related nature of condition management are need. *Application of universal and inclusive design principles:* The impairments commonly associated with older age should be considered during the design process to ensure technologies remain usable to all – application of universal design principles. *Adaptation to needs and requirements:* Where possible, individual customisation of technology interfaces, wearable locations or general appearance should be included in product design to enable adaptability to the needs of each user. Easy incorporation into the lives and homes of older adults. Multi-functional self-management to avoid the need for multiple technologies *Widening language requirements:* Apps and digital technologies should routinely be made available in multiple language options to enable use and carer support by different community groups. *Desirability and reduction is stigma:* Self-management technologies should be appealing in design and not contribute to disability stigma experienced by those living with long-term conditions. Aspirational. Avoid unappealing, clinical or stigmatising design that may leave the user feeling vulnerable. *Accuracy:* It is important to ensure the data used to underpin technologies that offer education, or advice is of the highest standard and where necessary can be updated when needed to reflect changing clinical narratives. *Careful design of updates:* Changes and updates to systems can be challenging for a user to navigate. The nature, frequency and impact of software updates that may change the general appearance of navigational pathways of digital systems should be careful considered and introduced. *Informed data use and sharing:* Emphasis should be placed on supporting the person to make informed decisions about who they wish to share their data with, rather than an assumption of open sharing with the clinical team.	*Training and support*: to ensure users have the appropriate skills and knowledge *TOngoing support packages:* Initial setting up / on boarding is crucial to trust in the technology. Detailed holistic assessment of the individual's needs and wishes. The development and tailoring of individualised support packages to ‘onboard’ or set up users, with consideration of ongoing technology support if needed to encourage long-term adoption. *Sufficient resource allocation:* The initial assessment and provision of self-management technologies to older adults should be considered as a complex intervention and therefore adequate time and resources should be allocated to the assessment and when necessary to re-assess needs. *Support for the wider circle of care:* Training, support and general information about the use of self-management apps and technologies should be given to family carers as well as to the older adult where appropriate, recognising their role in supporting both the management of long-term conditions and the use of the technology. *Informed data use and sharing:* Emphasis should be placed on supporting the person to make informed decisions about who they wish to share their data with, rather than an assumption of open sharing of data with the clinical team. *Maintain the momentum:* The development of local initiatives that build upon the uptake in digital technologies in older adults with long-term health conditions during the COVID-19 pandemic. *Adjustment for cognitive impairment:* When working with people who live with dementia and their family and carers, specialist communication strategies and additional support may be necessary to support use of self-management technology. *Ongoing support packages:* The development and tailoring of individualised support packages to ‘onboard’ or set up users, with consideration of ongoing technology support if needed to encourage long-term adoption. Multi-functional self-management to avoid the need for multiple technologies. Ongoing assessment of individual needs and support, with recognition of changing needs or wishes *Sufficient resource allocation:* The initial assessment and provision of self-management technologies to older adults should be considered as a complex intervention and therefore adequate time and resources should be allocated to the assessment and when necessary to re-assess needs.

## Discussion

There are a range of qualitative studies exploring the use of digital technologies to support self-management of CKD, diabetes and dementia, which informed the development of this modified e-Delphi study. We aimed to extend the condition-specific literature through an exploration of the challenges older adults face across the three long-term conditions and build a consensus of opinion among an expert panel about ways in which the design and the approach to providing technologies, might begin to address these challenges.

### A person-centred approach to provision and ongoing support of technology use

The role of the prescription and provision process in the use of digital health technologies has been identified and explored,^67,68^ but not to our knowledge, specifically across these three conditions. The need for person-centred assessment and provision^27,69^ when providing any form of intervention, especially digital technologies was emphasised by the panel. The results highlight the importance of the initial set-up and familiarisation experience, in developing confidence and trust in the technology, supporting appropriate use and encouraging long-term adoption. The findings imply that technologies are sometimes provided with limited, or no assessment of individual need, or follow-up, yet the needs and wishes of an older adult are individual and may change over time. Whether a piece of technology is purchased privately by the user or their family, or prescribed though healthcare services, its provision and ongoing support needs careful planning.

Across conditions, it was evident that the future allocation of resources should enable initial training, ongoing support packages and regular re-assessment to older adults. Building on current approaches to creating a better match between the person and technology^43^ further condition-specific research and guidance is needed to support clinicians (e.g. specialist nurses, occupational therapists, dieticians) in selecting and supporting the use of self-management technologies in relation to the condition, individual characteristics and in respect to multi-morbidity.

### The role of healthcare works and caregivers

Our findings draw attention to potential skills gaps among healthcare workers. This echoes the Topol Review^42^ that offers recommendations regarding the use of innovative technologies to improve NHS services. Further action is required to identify and meet the specific digital, prescription and support skills gaps in the health and social care workforce and to ensure confident provision and ongoing follow-up support and advice to older adults.^42^

The value of informal care is significant in the introduction and adoption of self-management technology,^70^ Our findings recognise and embrace the role of the wider circle of care, in access to, acceptance of and the adoption of technology. The panel advocated the inclusion of a wider network of people and specifically family and carers, in the provision of and training in the use of technology, while carers are often acknowledged as playing a critical role in long-term condition management,^29,31^ they may themselves be older adults, with their own specific needs to be met in order to effectively support another person's use of self-management technology. There is more to be done therefore, to optimise the support informal carers can provide, whist ensuring they are fully supported and enabled.

While some older adults benefit from a network of social support post provision, others are left to manage independently. The interviews, in line with a growing body of literature^71–73^ highlighted the role peer support groups may also play in the provision and ongoing effective use of technology.

### Managing health data

This study has drawn attention to older adults’ concerns around health-related data in respect to digital self-management technology. There are various standards to guide practice^74^ and the concern has been highlighted elsewhere.^75,76^ Here the entry, management and sharing of health data to support condition management featured strongly. It was felt that the perceived security and trustworthiness of the data collected, stored and potentially shared, affected overall trust in technology. Furthermore, the interviews drew attention to the cognitive load and potential psychological impact of collecting, viewing and interpreting health data and the need for support in data interpretation (e.g. recognising and responding to a decline in one's condition). The importance of control and choice were also emphasised. These elements are all important to consider, but raise challenges in how design can be employed to enable understanding, transparency, choice and control for all older adults.

### The digital divide

The digital divide continues to affect access to care and individual capacity to self-manage with the assistance of technology.^9,12,39,77^ The interviews in round 1 highlighted that while digital uptake and digital skills, increased during the pandemic, digital access and literacy among older adults is variable. They pointed to variation in access to self-management technologies across the UK, further exacerbated by socio-demographic factors, variation in technology infrastructure (e.g. reliable Internet services), lack of access to state and specialist services and language barriers. The results placed a particular emphasis in the panel's experience, on the role of poverty (e.g. on accessing and maintaining the use of technologies and smartphone). This supports wider discussion on how poverty affects access to healthcare and health technology and how the digital divide can exacerbate health inequalities, with targeted efforts required to better engage, invest and develop new approaches to improve access for all.^78,79^

In support of others, calling for greater representation in participant and public involvement activities^80^ and design,^81^ among our experts, there was strong agreement that technology development, research and provision in the UK does not adequately include a diverse and representative group of users. The interview findings pointed to the need to improve involvement in respect to language, cultural background, personal income, physical and cognitive capacity and access to social and technical support to achieve a more inclusive design language and provision.

### Designing for the individual as they age

Our findings reaffirm research arguing that the available technologies can lack appeal for older adults for a variety of reasons^82^ including appearance, ease of use and failure to take into account the specific needs and capabilities of the user. The need for good usability is well recognised^83^; here we emphasise the importance of usability in some key areas for example in the understandability of ‘behind the scenes’ operations such as data usage and management and the installation of updates. These elements may not be at the forefront of the design process, but their simplicity and transparency affect the sense of trust, user control and empowerment.

The panel did not reach consensus in respect to customisation at the point of technology provision, however they did agree that design should avoid a ‘one size fits all’ solution and that self-management technologies should be aspirational and adaptable to the lives of individuals. Design should counter stigma, both in the need to use a specific piece of technology to support health and well-being and associated with age and age-related changes in capabilities.

There has been an increased focus on empathy, inclusion and accessibility in design research and practice,^84–87^ but the development and testing of medical and healthcare technology often still focuses on functionality and clinical benefit, neglecting the wider psychosocial factors that influence whether the technology will be used effectively in practice by older adults as they age.^88^ We argue for a stronger focus on design for the individual as they age, taking into account the social, physical, cognitive and emotional factors affecting uptake and usage of technology, many of which may change with age. There is a need for more desirable technologies, which are functional and able to support condition management and progression, while also catering for age-related capabilities and being easily incorporated into the lives and homes of older adults with long-term health conditions.

### Design for integrated and inter-related condition management

Alongside an increasing body of research exploring multi-morbidity and inter-related conditions^1,13,21,30^ there is limited evidence of research focusing on the shared requirements across the three conditions considered here. When living with more than one condition, there is increased complexity and demands from monitoring and managing multiple sets of symptoms and treatment regimes, the need for different data capture and presentation and interaction with multiple clinical specialities. This presents significant complexity for not just the user, but also the clinician and the designer. Technology supporting self-management of more than one condition potentially enables shared functionality, but may add unmanageable complexity to the information requirements and demands on the user. Further research is needed to consider the requirements of people with multiple long-term conditions to inform the development of future technologies that can offer multiple functions and ‘joined up’ data collection, while not overburdening the user.

Arguably, design in this context requires a nuanced understanding of the characteristics of the individual condition, the impact of multi-morbidity and the need to adapt to co-occurring and progressive conditions such as dementia. This is a complex set of requirements, but by building understanding across inter-related long-term conditions and through collaborative, multi-disciplinary approaches, we may be better placed to design, identify and provide suitable solutions.

### Future directions

Through this study, we have sought to build understanding and agreement on how technology design and prescription might be enhanced to support the condition management of three long-term conditions prevalent among older adults. We have considered three conditions that fall within the remit of the NIHR Devices for Dignity HealthTech Research Centre (D4D). Hosted within the NHS, D4D works with national collaborative networks (involving patients, carers, healthcare professionals, industry, charities and academia) to catalyse and co-create safe and usable devices and technology-dependent interventions for diverse chronic or life-long health conditions typically associated with loss of dignity and independence. This study informs an ongoing programme of research and practice in these areas, considering some of the needs and challenges that are common to older adults affected by the three conditions. The consensus developed here and the emerging questions (summarised in Table 5) will guide a wider programme of work to support the use of self-management technology by those living with long-term conditions.

**Table 5.** Summary of emerging research questions.

	Directions for future research
1	Which strategies should be prioritised to overcome common barriers to self-management technology use by older adults, enabling those with limited private funds, or limited technology skills?
2	Which specific and/or diverse groups (clinical and demographic) within UK communities would most benefit from the use of self-management technologies to manage their long-term health conditions? What are the unique barriers to use and access to technologies for these groups?
3	Building on the Topol review and in relation to this study, research should consider: What are the specific digital skills gap in the health and social care workforce that should be addressed to enable staff to be confident providers and advisors of assistive technologies for all?
4	What are the technology needs of family carers who use self-management technologies to support an older person with a long-term condition? What are the particular needs of those caring for people living with dementia?
5	How can online peer support groups built into the provision of self-management technologies and patient and carer education programmes be further developed to meet condition specific and demographic specific needs and thereby benefit the management of long-term health conditions?
6	How can we co-create future technologies for people with multiple long-term conditions so they offer multiple functions and ‘joined up’ data collections, while not overburdening the user?
7	How can we address issues of trust in new self-management technologies and regaining trust where users have previously had poor experiences?
8	How can design be employed to reduce the impact of poverty on accepting and maintaining the use of technologies?

### Limitations

The data presented may be considered limited by the small number of panel experts involved. The Delphi method seeks to reach consensus among experts rather than achieve generalisation of results to a larger population, thus a Delphi panel often includes 10–18 experts.^89^ The group of 14 was manageable in size at a time of high demands on healthcare due to the pandemic and the composition provided a range of different experiences and subject expertise. The size and make-up of the panel did limit our ability to analyse the data per participant group and, ideally, the panel would have included more older adults, at least one panel member with lived experience of dementia, as well as carers.

The e-Delphi method was modified through the use of interviews in round 1.^90^ This modification was a useful first step to validate and expand on the findings from the literature review and guide the online data collection. The data was collected via online methods due to restrictions related to the COVID-19 pandemic affecting the ability to bring participants together, particularly those with lived experience of a long-term condition, or working closely with them, but allowing the geographically distributed and participants who were isolating, to take part. The pandemic arguably accelerated the uptake and use of digital technologies in healthcare and undertaking the study at this time, provided the context for exploring the potential increase use of technology for self-management, alongside the panel's heightened awareness of the patient groups who found access and digital care most challenging.

Our approach involved the panel considering their experience of a specific condition, or technology use by that patient group. They were not asked to consider specifically the scenario of multi-morbidity and inter-related conditions, rather the focus was on drawing commonalities through the analysis. The next step of the research will be to focus on the specific patient requirements of managing multiple conditions and the implications for the design and provision of technology for patients affected by interrelated long-term conditions.

## Conclusions

A modified e-Delphi method was used to explore and agree on common enablers and barriers to the use of digital technology by older adults for the self-management of three chronic conditions (diabetes, dementia and chronic kidney disease). The approach has enabled consensus from an interdisciplinary panel of experts. The shared knowledge across conditions has led to suggestions for future research and development related to digital self-management technology, as well as recommendations to guide those designing and providing digital self-management technologies to older adults. It is concluded that a person-centred approach that focuses on the individual as they age and takes into account the social, physical, cognitive and emotional factors affecting the uptake and usage of technology is needed to advance acceptance, use and long-term adoption by older adults.
